# The pursue of quality

**DOI:** 10.1590/2177-6709.20.5.014-014.edt

**Published:** 2015

**Authors:** Eustaquio Araujo

**Affiliations:** Former president of the Brazilian Board of Orthodontics and Dentofacial Orthopedics; professor and vice-director of Center for Advanced Dental Education; clinical director of Orthodontics Program at Saint Louis University, USA.

Life goes on and maturity arrives with aging and makes us more sensitive. This is a unique time in our lives and people consider it "experience"; however, wouldn't "common sense" be a better definition? Our lives are absorbed by our work and dedication to our profession. As leaders and role models, it is our responsibility to demonstrate work ethics, devotion and dedication. 

Allow me to go back in time and place myself in the shoes of our young colleagues - recent graduates or those about to graduate in Dentistry - who normally are full of plans and dreams. New ideas are popping up and great plans are made for the future. At this point, however, they face the unfair reality of the real world (the market) and are approached by unscrupulous "*sellers of illusions*" with false promises of "*making them orthodontists*" within a brief amount of time and opening the doors of the future to them.

They dream more and more, they undertake serious financial responsibilities and certainly become the new victims of an immoral system surrendering themselves to a "*street corner market*", to the vendors of professional education. 

Statistical data do not lie. Brazil is the country with the highest number of graduate orthodontic courses in the world. A few years ago, there were 600; at present, there might be around 1000. Yet, how many of these courses do really qualify these professionals? It is hard to tell.

This "*cheap*" education has spread so rapidly that it undermines our reputability and respect as professionals. The media makes fun of "*the braces craze*", a fashion taken up with enthusiasm by many, and maybe inadvertently creates TV humor sketches to denigrate our specialty. 

My reality has been, for many years, quite different, but although I have lived a different experience, I continue to fight for Brazilian Orthodontics, especially when confronted with some pejorative comments I hear around the world.

But let's leave this issue aside and focus on the good and positive. What is our current status? I assure you that should there be a podium for world Orthodontics, Brazil would certainly be on it. I am proud to see the work of many Brazilian colleagues when presenting in national and international congresses, as well as the many contributions to the literature with articles/ research/case reports published in the most renowned orthodontic journals. I advocate the quality of our contributions. Many colleagues stand out in the crowd, and we wish this number continues to increase. We have made a lot of progress and attained our current status thanks to the work of many. 

This proves that we are capable of doing it and that it can be well done. Why not making this our main cause? In order to do so, it is important to highlight that we need a parameter for the necessary qualification or our specialists. 

I have just finished my tenure as president of the Brazilian Board of Orthodontics and Dentofacial Orthopedics (BBO). During approximately 8 years, I had the chance to work with and learn from many leaders of our profession; colleagues whose principles, aspirations and objectives are very similar to mine. I also met many young courageous and talented professionals deeply concerned with excellence and quality. Maybe this is the path to take.

On behalf of many colleagues certified by the BBO and my own, I propose and offer the Brazilian Board of Orthodontics and Dentofacial Orthopedics as the standard to be followed towards the so longed-for excellence. Unfortunately, by law, our country only allows Law professionals to sit for official board qualification examinations (the Brazilian Bar Association exam). All attempts made to allow health professionals to create similar exams have been unsuccessful. Nevertheless, the BBO has undoubtedly and independently, under the umbrella of ABOR, tried to fill this gap. Yes, we are able to do it with quality.

My own experience with board examination is a good example. I decided to sit for BBO exam, when I was maybe a little "old" to do so, but I only gained from it; as it made me a better orthodontist and human being. I call upon all my colleagues and professors who deal with the dreams of many youngsters to fight for them and with them. In October, 2015, Phase I examination will be held in the city of Florianópolis during the 11^th^ ABOR Congress. In March, 2016, also in Florianópolis, both Phases I and II will be offered again. Please explore and enjoy our website (www.bbo.org.br) to have a better idea of our goals and guidelines.

This is an invitation; better yet, a request to act as protagonists in defense of quality and excellence in our specialty. 

We are not identical, but we can dream the same dreams while focusing on the beauty and art of our specialty and treating our patients with respect, giving to others what we wish for ourselves: quality of life.

